# Sleep, energy disturbances and pre-competitive stress in female traveller athletes

**DOI:** 10.5935/1984-0063.20190093

**Published:** 2019

**Authors:** Maria-Raquel G. Silva, Teresa Paiva

**Affiliations:** 1 Faculty of Health Sciences, University Fernando Pessoa, Oporto, Portugal. Research Centre for Anthropology and Health, University of Coimbra, Coimbra, Portugal. Scientific Commission of the Gymnastics Federation of Portugal, Lisbon - Portugal.; Research Centre for Anthropology and Health, University of Coimbra, Coimbra, Portugal.; Scientific Commission of the Gymnastics Federation of Portugal, Lisbon - Portugal.; 2 CENC, Sleep Medicine Center, Sleep Medicine - Lisbon - Portugal.

**Keywords:** Sleep, Stress, Psychological, Energy, Athlete, Travel

## Abstract

**Background:**

Gymnasts of high performance level are submitted to a demanding competitive schedule, which can negatively affect their circadian rhythm, sleep, appetite and pre-competitive stress. Therefore, the purpose of this study was to investigate sleep, body composition, pre-competitive stress and energy in elite female athletes just before a World Cup and potential sleep risks according to the travelled distance by athletes in order to compete.

**Methods:**

Sixty-seven rhythmic gymnasts of high performance level were evaluated in order to collect training and competition data, daytime sleepiness (DS) by the Epworth Sleepiness Scale, sleep quality (SQ) by the Pittsburgh Sleep Quality Index, precompetitive anxiety by the Sport Competition Anxiety Test-A and dietary intake prior to a World Cup.

**Results:**

Gymnasts suffering from severe DS had worse scores in the competition than those who reported normal DS (*p*=0.004). In addition, gymnasts with severe DS reported reduced SQ (*p*=0.014) and showed high levels of precompetitive stress (*p*<0.01). Gymnasts with reduced SQ demonstrated great DS (*p*=0.014) and high levels of precompetitive stress (*p*=0.010).

**Conclusions:**

Gymnasts demonstrated disordered sleep and precompetitive stress. Athletes who travelled long distances to compete presented high risk for short sleep duration, abnormal DS and reduced SQ.

## INTRODUCTION

Sleep is usually regarded as an important resource for the physical and mental well-being of any athlete. Indeed, it is crucial for athletic performance and recovery from training sessions, competition events or transmeridian travels^1^^,^^2^. It also reduces the risk of developing a state of overtraining^1^^-^4. Sleep is a complex physiological and active state that is divided into two major states, namely non-rapid eye movement (NREM) and rapid eye movement (REM) sleep. The first state is characterized by four stages linked to a progressive increase in sleep’s depth^5^. The REM sleep is associated to muscle atonia and dreaming^1^. The deep sleep, in particular the slow-wave sleep, is related to recovery by the synchronization of the growth hormone release with the slow-wave sleep, promoting optimal conditions for anabolic reactions^6^. However, in case of sleep deprivation by a reduction on the slow-wave sleep, a decrease in performance and an increase in daytime sleepiness (DS) and in cortisol concentration have been reported^7^^,^^8^.

In fact, athletic performance in competition and stress have been negatively correlated with DS and sleep quality (SQ)^4^^,^^9^. Chronic physical exercise, defined as one that significantly modifies somatic functions as long-term improvements, is considered to have a great effect on improving SQ, since it reduces both sleep latency and waking time during sleep, and increases total sleep duration^10^^,^^11^. Indeed, chronic physical exercise improves body composition, appetite regulation, basal metabolic rate, cardiac function, glycemia, and immune function^10^. It also promotes mood and regulates exposure to stress and its effects, which improves the sleep pattern^10^. Although the practice of regular physical exercise may increase the NREM sleep activity, its practice shortly before bedtime can generate a stressful effect by reducing the amount of NREM sleep^10^, that is why, it is not advisable for athletes to train or compete at night (>8:00pm)^12^^,^^13^.

Apparently, individual sports’ athletes have more sleep problems and anxiety than team sports’ athletes^13^^,^^14^. On the other hand, team sports have a more regular competitive schedule and athletes can better develop pre-competitive routines, while individual sports have a more irregular competitive schedule, and the athlete may not compete for weeks^6^. Although sleep is considered an important resource for athletes’ success^13^^,^^15^, and more research has been made, published studies on athletes’ sleep before competitions^4^ are fewer than during training periods.

Gymnasts of high performance level are submitted to a competitive schedule that does not facilitate the regulation of various functions related to sleep, appetite and the dark-light cycle due to travels carried out inter-competitions^1^^,^^4^^,^^16^. In addition, Schaal et al.^17^ concluded that athletes of both sexes, who practice aesthetic sports, are the most affected by anxiety, when compared to those who practice other sports.

Although female athletes are less studied than male athletes, and even fewer studies have been investigated about sleep in females, a recent one with elite female gymnasts highlighted that athletic performance was positively associated with sleep duration and negatively associated withDS, SQ and energy availability (EA)^4^. Therefore, the purposes of this study were to investigate sleep, body composition, pre-competitive stress, and energy in elite female athletes just before an international competition and potential sleep risks according to the travelled distance by athletes in order to compete.

## MATERIAL AND METHODS

### Participants

Sixty-seven rhythmic gymnasts (18.7±2.9 years old) of high performance level were evaluated in order to collect training and competition data, DS and SQ, precompetitive anxiety and dietary intake before 1 to 4 days of the *Fédération International de Gymnastique* World Cup and the Rhythmic Gymnastics International Tournament in 2011. Gymnasts were recruited through personal contacts or through their coaches and volunteered to participate. The response rate was 58.2%. Study design was approved by the Ethical Committee of Medical Faculty of Lisbon (01.06.2011CHLN/HSM/HPV/IMM) and written informed consent was obtained from all participants.

### Training and competition data

The number of training sessions per week and the number of hours of training sessions per day allowed calculation of the number of training hours per week. Performance was examined using the overall performance ranking of each participant from the published final list of general competition results.

### Body composition

Body mass (BM) was measured by a digital scale (SECA-872, Hamburg, Germany) to the nearest .01 kg wearing T-shirt and gym shorts before the warming up session. Height was determined with a portable stadiometer (SECA-213, Hamburg, Germany) to the nearest 0.1cm. Procedures were conducted as recommended by the International Society for the Advancement of Kinanthropometry^18^. Body mass index (BMI) was calculated as a ratio of weight to the squared height (kg/m^2^). Body fat (BF), fat-free mass (FFM) and total body water (TBW) were assessed by bio-impedance analysis (TANITABC-545, UK), which can provide fairly accurate estimates of FFM and TBW^19^.

### Sleep

Bed time and awake time during the week and at weekends were obtained together with subjective sleep duration. Variability was measured by the difference in sleep duration during weekends and week days.

Daytime sleepiness was measured by the Epworth Sleepiness Scale (ESS)^20^ and SQ by the Pittsburgh Sleep Quality Index (PSQI)^21^. The total ESS score can range from 0 (zero) to 24 points. A score between 0-9 points is matched as no DS; between 10 and 12 points, mild sleepiness; between 13 and 16 points, moderate sleepiness and; above 17 points, severe sleepiness^20^. The PSQI score ranges from 0 (zero) to 21 points. A total score equal to or less than five points is associated with a good SQ and the total score above 5 is considered poor SQ^21^.

### Precompetitive anxiety

The Sport Competition Anxiety Test form A (SCAT-A) or Illinois Competition Questionnaire was applied. SCAT-A was developed by Martens^22^ to evaluate the trait anxiety in a sport event, generally defined as the pre-competitive anxiety; it consists of 15 items, with responses classified as rarely, sometimes and often. A score less than 17 points is a considered reduced level of stress; a score between 17 and 24 points is a moderate level and a high level of stress whenever the score is higher than 24 points.

### Energy assessment

Participants were asked to record all foods and beverages typically consumed for the 24 hours before the interview, including time of day and meal type. Foods were expressed in household measurements and converted to grams and millilitres for a quantitative analysis of energy intake (EI). The basal metabolic rate (BMR) was calculated using the Cunningham equation, as suggested by the American College of Sports Medicine^19^.

Although the Cunningham equation has been used to determine energy expenditure rate in several sport-based studies, and laboratorial facilities were not available to evaluate the athletes of our study, this equation was only used to estimate BMR, since numeric guidelines such as this provide an approximation of the average energy expenditure of an individual athlete^16^. Energy availability (EA) was estimated^16^; low energy availability (LEA) was defined as EA<45kcal/kg FFM/day; and a threshold below 30 kcal/kg FFM/day was also investigated, since it is considered the lowest energy threshold of EA for women^19^. Exercise energy expenditure (EEE) was calculated using the 2011 Compendium of Physical Activities^23^. These calculations accounted for exercise duration, the intensity of the gymnastics training and BM, which were collected using a characterization questionnaire.

### Statistical analysis

The characteristics of the participants are described with proportions for categorical variables and with mean and standard deviation values for continuous variables. Spearman correlation coefficient was used to determine associations between categorical and continuous variables; due to the number of subjects evaluated, the significance level used was 1% (*p*<0.01). Bivariate correlations were run on continuous measures of demographics, body composition, EI, ESS, PSQI, anxiety and performance. To identify sleep predictors (sleep duration, DS and SQ) related to distance travelled to compete, logistic regression analyses were performed with “0” for distance ≤5000km and “1” distance>5000km. Sleep corresponding cut-offs were defined as follows: short sleep duration≤08h30^24^^,^^25^; abnormal DS for ESS>9^20^ and; reduced SQ for PSQI>5^21^. Unadjusted odds ratios (OR) and 95% confidence intervals (CI) were calculated using univariate logistic regression. The significance level was 5% (*p*<0.05). Data was analyzed using IBM SPSS statistical software version 25.0 for Windows (New York, USA).

## RESULTS

Gymnasts were training a mean of 36.6±7.6 hours per week and reported 11.5±3.2 years of Rhythmic Gymnastics’ experience.

Participants’ BM (48.4±4.9kg) and BMI (17.4±1.1kg/m^2^) were below the normal for age (10^th^ to 50^th^ percentiles) and height (1.66±0.05m) was normal to slightly above normal for age (50^th^ to 75^th^ percentiles, Table 1). The gymnasts’ BF was below the estimated minimal value compatible with health for female athletes (Table 1); 37.3% of gymnasts presented EA below 45kcal/kg FFM/day and 44.8% demonstrated EA below 30 kcal/kg FFM/day. Approximately 19.4% of the athletes presented high levels of precompetitive stress.

**Table 1:** Age, training, menarche, anthropometric characteristics, sleep, precompetitive stress and energy of the participants (n=67).

Variables		n	%	Mean±SD	Range
Age (years)	-	-	-	18.7±2.9	16-26
Years of practice	-	-	-	11.5±3.2	10-18
Training (h/week)	-	-	-	36.6±7.6	25-54
BM (Kg)	-	-	-	48.4±4.9	36-55
Height (m)	-	-	-	1.66±0.05	1.51-1.73
BMI (Kg/m^2^)	-	-	-	17.4±1.1	14.9-20.2
BF (%)	-	-	-	9.0±2.2	6-16
FFM (Kg)	-	-	-	28.2±4.6	20-40
Sleep duration, week days	-	-	-	8h10±1h30	6h00-9h30
Sleep duration, weekend days	-	-	-	8h23±1h36	7h00-9h00
ESS global score	-	-	-	10.2±3.1	6-18
	No DS	45	67.2	7.8±0.6	6-8
	Mild sleepiness	13	19.4	9.5±0.9	9-12
	Moderate sleepiness	0	0	--	--
	Severe sleepiness	9	13.5	17.6±0.5	17-18
PSQI global score					
	Good quality	15	22.4	3.5±1.0	2-5
	Poor quality	52	77.6	8.0±1.8	6-12
Sleep duration at week				8.1±1.3	6-9
	<8 hours	38	56.7	7.0±0.4	6-7.4
	8-9 hours	22	32.8	8.3±0.1	8-8.5
	>9 hours	7	10.4	9.3±0.0	--
Sleep duration at weekend				8.3±1.1	7-9
	<8 hours	24	35.8	7.0±0.1	7-7.3
	8-9 hours	43	64.2	8.4±0.4	8-9
	>9 hours	0	--	--	--
SCAT-A				22.7±3.2	13-30
	Reduced level	1	1.5	13.0	--
	Moderate level	53	79.1	21.8±2.1	19-24
	High level	13	19.4	26.9±2.5	25-30
EI (kcal/day)				1709.6±329.7	979-2320
BMR (kcal/day)				1137.4±112.3	937-1384
EEE (kcal/day)				789.9±243.7	445-1369
EA (kcal/kg FFM/day)				31.5±11.9	26-59
	≤30kcal/kg FFM/day	30	44.8	29.1±8.9	19-31
	≤ 45kcal/kg FFM/day	25	37.3	33.5±12.6	28-40
	>45kcal/kg FFM/day	12	17.9	46.7±11.0	36-49

BF: Body fat, BM: Body mass, BMI: Body mass index, DS: Daytime sleepiness, EI: Energy intake, ESS: Epworth Sleepiness Scale, FFM: Fat-free mass, PSQI: Pittsburgh Sleep Quality Index, SCAT-A: Sport Competition Anxiety Test form A.

**Figure 1:**
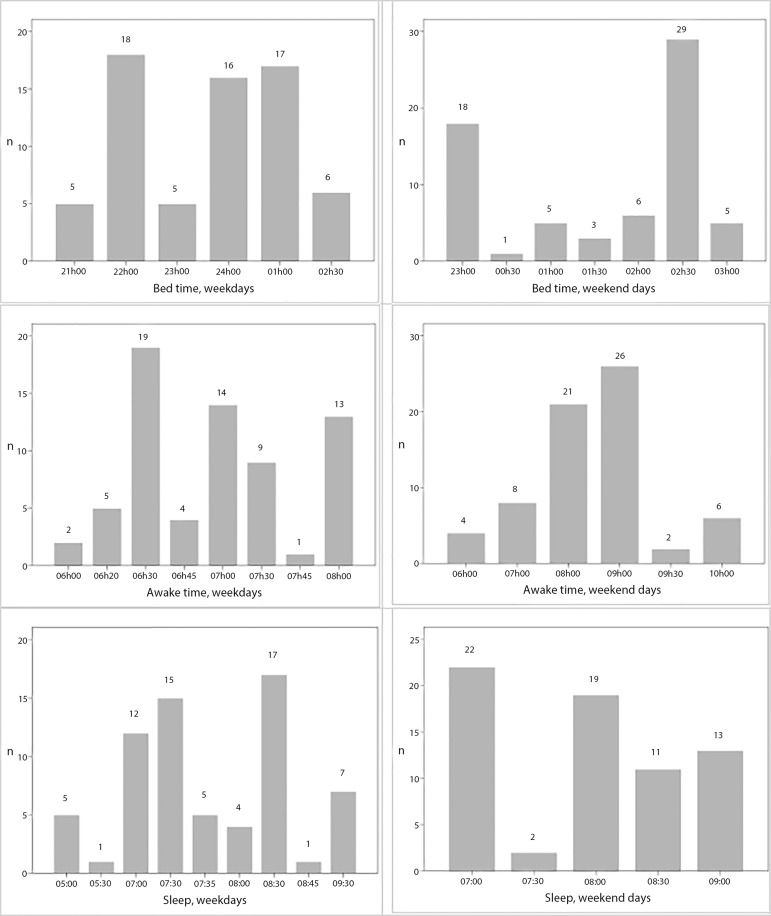
Bed time, awake time and sleep duration on weekdays and weekend days of participants (n=67).

The mean sleep duration on weekdays was 8h10±1h30min and most gymnasts (56.7%) slept less than 8 hours (Figure 1). On weekends, most athletes (64.2%) presented an appropriate duration of sleep and 35.8% slept less than 8 hours (Figure 1).

**Figure 2:**
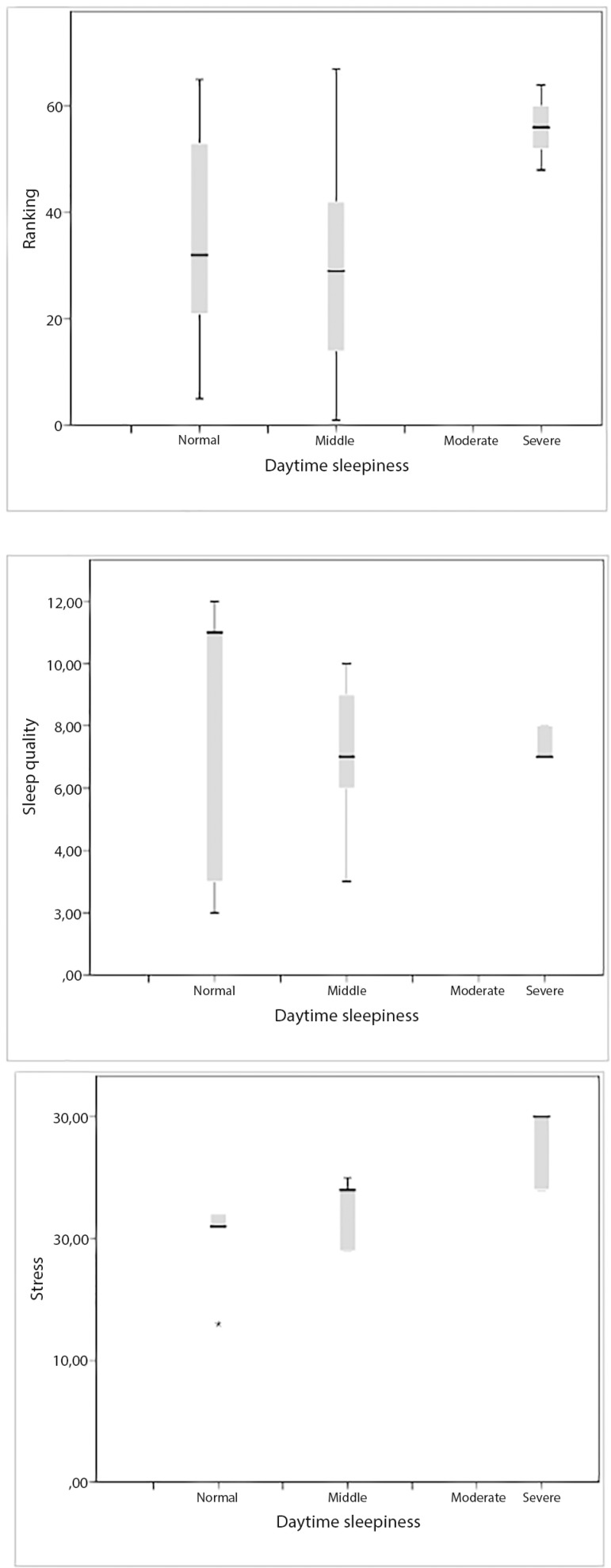
Association between daytime sleepiness with ranking (a), sleep quality (b) and pre-competitive stress (c) of the participants (n=67). *p≤0.01.

The average score for the ESS was 10.2±3.1 (Table 1); most athletes (67.2%) showed no DS, from which 32 gymnasts (47.8%) were immediately below the threshold of mild DS. In addition, 9 athletes (13.4%) suffered from severe DS and 13 (19.4%) suffered from middle DS. Gymnasts suffering from severe DS had worse scores in the competition than those who reported normal DS (*p*=0.004, Figure 2a). In addition, gymnasts with severe DS reported reduced SQ (*p*=0.014, Figure 2b) and showed high levels (> 24 points) of precompetitive stress (*p*<0.01) (Figure 2c).

**Figure 3:**
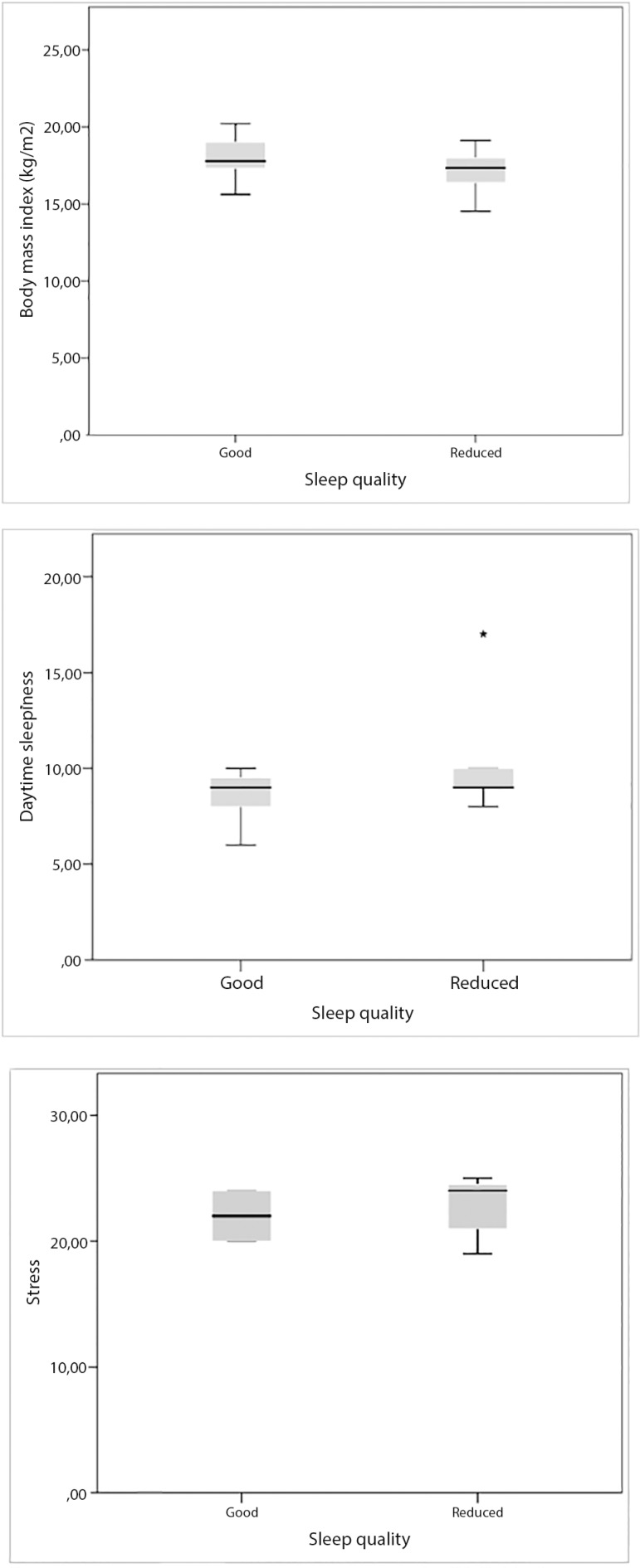
Association between sleep quality with BMI (a), daytime sleepiness (b) and pre-competitive stress (c) of the participants (n=67). *p≤0.01.

The average PSQI score was 7.0±2.54 (Table 1); most gymnasts (77.6%) had reduced SQ. Athletes with good SQ had a BMI of ≥18.5 kg/m^2^, in contrast to those who had a poor SQ, whose BMI was below normal (<18.5 kg/m^2^, *p*=0.005; Figure 3a). Gymnasts with reduced SQ demonstrated great DS (*p*=0.014, Figure 3b) and high levels of precompetitive stress (*p*=0.010, Figure 3c).

Although our participants were from several nationalities (North and South America, Asia, Europe and Oceania) and travelled across different time zones to compete in an environment that may be both geographically distant and different from the home-base, there was no association between the participants’ athletic performance and their country of origin. However, there were risk factors associated to the gymnasts’ travelled distance, as follows: gymnasts who travelled more than 5000 km in order to compete presented a higher risk for short sleep duration (OR=6.52, *p*=0.041), abnormal DS (OR=6.34, *p*=0.030) and reduced SQ (OR=12.28, *p*=0.021) than those who travelled equal or less than 5000km to compete (Table 2).

**Table 2:** The descriptive analyses and logistical analyses for sleep risk factors regarding the distance travelled by gymnasts (n= 67) in order to compete.

	Distance travelled for competition ≤5000 km (n=40)			Distance travelled for competition >5000 km (n=27)			
	Mean±SD	n (%)	OR (95% CI)	Mean±SD	n (%)	OR (95% CI)	**p**
Short sleep duration (n=51)	6:20±1:04	24 (60%)	1.81 (0.69-2.64)	5:57±1:19	27 (100%)	6.52 (1.76-18.62)	0.041*
Abnormal DS (n=22)	11.5±1.2	12 (54.5%)	4.29 (3.16-5.02)	17.5±1.6	10 (45.5%)	6.34 (2.01-8.70)	0.030*
Reduced SQ (n=52)	7.9±3.1	25 (48.1%)	9.03 (8.06-12.35)	10.6±2.8	27 (51.9%)	12.28(10.74-17.02)	0.021*

## DISCUSSION

The general finding from sleep medicine that international athletes can suffer from sleep disturbances is confirmed by our results.

Gymnasts who suffered from severe DS prior to competition had worse scores in competition than those who reported normal DS. In addition, gymnasts with reduced SQ demonstrated great DS and high levels of precompetitive stress.

Since sleep was evaluated prior to a world competition, it is possible that athletes may underwent to the so-called “first night effect”^13^ and/or were negatively influenced by some circadian disruption or jet-lag effect due to the distance travelled in order to compete^15^. Although not evaluated, prolonged sleep latency due to difficulties in falling asleep and frequent awakenings at night due to difficulties in maintaining sleep, in addition to waking up early in the morning and not feeling invigorated in the morning are indicators of non-restorative sleep^11^^,^^13^^,^^26^^-^31, typical of a partial restraint of sleep before a competition^13^, which may have influenced DS and SQ of our participants. In addition, and due to the fact that gymnasts who travelled longer distances to compete presented a higher risk for short sleep duration, abnormal DS and reduced SQ than those who travelled shorter distances, it is possible that the first probably had more difficulty to resynchronise their circadian rhythm^24^^,^^26^ or the acclimatization time at the new time was insufficient or insufficiently planned^25^.

In addition, high burden of responsibilities during this international competition may have affected gymnasts’ pre-competitive stress, which can also be influenced by a disordered sleep as shown by our results.

In a study conducted by Erlacher et al.^13^ with 225 athletes from team sports and 407 athletes from individual sports observed that 65.8% of athletes already had a reduced SQ at least once on the previous night to a major competition, and 62.3% had this experience at least once in the 12 months prior to the study. This phenomenon, called “first night effect”, reduces SQ during the first few nights at an unknown location. Schaal et al.^17^ suggest that girls may be more sensitive to the effects of stress and experience more behaviors related to depression and anxiety than boys. Buysse et al.^32^ found higher insomnia rates and more perceived negative effects of insomnia in women than in men. In our study, no data about gender differences was available, since Rhythmic Gymnastics is an exclusive female sport, which means that our participants may be vulnerable to disordered sleep. On the other hand, some studies^13^^,^^14^ failed to conclude that female athletes have more problems with sleep than males.

In addition to sport and competition demands, and participants’ mean age, our gymnasts may be close to an early onset sleep, which was defined by Hagenauer et al.^33^ at around 19.5 years old. Carskadon^5^ argues that adolescents have a greater resistance to sleep pressure, which allows them to stay up later than prepubescent children. Jenni et al.^34^ explained this aspect by stating that the construction of sleep pressure was slower in the post-pubertal than in the pre-pubertal period. In fact, adolescents are more intransigent to the light response in the morning, which affects the time of waking up and have an exaggerated response of the phase delay due to exposure of night light, affecting the sleep pressure to and increasing the capacity to stay awake^35^. Older adolescents take longer to fall asleep and are more able to stay awake than younger teens^36^.

There are very few studies that evaluated the effect of traveling long distances on sports performance^37^^-^40. The existing studies have shown that performance levels decrease with jet-lag^15^. It appears to be the result of physiological desynchronization and sleep disturbances, leading to suboptimal blood pressure, heart rate, body temperature, and muscle strength. One recent published study^4^ concerning the precompetitive sleep in elite female gymnasts has demonstrated that athletic performance in an international competition was positively correlated with sleep duration and negatively correlated with SQ and DS. In addition, another published research^9^ with female gymnasts has shown that age, training regime, menstruation, individual preferences for bedtime, body composition and energy were predictors of gymnasts’ precompetitive sleep with consequences upon their sleep duration, SQ and DS, but no sleep risks considering the distance travelled prior the competition were investigated until this study.

Therefore, it is important to highlight that although sleep helps the athlete’s recovery and that, in turn, generates the promotion of a feeling of well-being in the next day and SQ^15^, traveling long distances prior to competition affects sleep in quantity and quality and inhibits that feeling of well-being through DS. We should emphasize that sleep supplementation with napping is important, especially in traveler athletes, given its positive effect on the cognitive component of behavior related to learning skills, strategy and tactics^41^.

In summary, rhythmic gymnasts with reduced SQ demonstrated great DS and high levels of precompetitive stress. Gymnasts suffering from severe DS had worse scores in the competition than those who reported normal DS, and showed high levels of precompetitive stress. Athletes who travelled longer distances to compete presented a higher risk for short sleep duration, abnormal DS and reduced SQ than those who travelled shorter distances.
